# Roles and barriers of community pharmacy professionals in the prevention and management of noncommunicable diseases in Ethiopia: a systematic review

**DOI:** 10.3389/fpubh.2025.1485327

**Published:** 2025-08-28

**Authors:** Ashenafi Kibret Sendekie, Eyayaw Ashete Belachew, Liknaw Workie Limenh, Gashaw Sisay Chanie, Gizachew Kassahun Bizuneh, Abera Dessie Dagnaw, Yabibal Berie Tadesse, Kalab Yigermal Gete, Fasil Bayafers Tamene, Biruk Beletew Abate

**Affiliations:** ^1^Department of Clinical Pharmacy, School of Pharmacy, College of Medicine and Health Sciences, University of Gondar, Gondar, Ethiopia; ^2^Curtin Medical School, Faculty of Health Sciences, Curtin University, Bentley, WA, Australia; ^3^Department of Pharmaceutics and Social Pharmacy, School of Pharmacy, College of Medicine and Health Sciences, University of Gondar, Gondar, Ethiopia; ^4^Department of Pharmacognosy, School of Pharmacy, College of Medicine and Health Sciences, University of Gondar, Gondar, Ethiopia; ^5^Department of Pharmaceutical Chemistry, School of Pharmacy, College of Medicine and Health Sciences, University of Gondar, Gondar, Ethiopia; ^6^School of Medicine, College of Medicine and Health Science, Bahir Dar University, Bahir Dar, Ethiopia; ^7^Department of Pharmacy, College of Health Sciences, Debre Markos University, Debre Markos, Ethiopia; ^8^College of Medicine and Health Sciences, Woldia University, Woldia, Ethiopia; ^9^School of Population Health, Curtin University, Bentley, WA, Australia

**Keywords:** community pharmacy professionals, community pharmacy, roles, barriers, noncommunicable diseases, Ethiopia

## Abstract

**Background:**

Pharmacists play a crucial role in the prevention and management of noncommunicable diseases (NCDs) by providing medication therapy management, disease monitoring, and patient education. However, existing evidence on the roles and barriers of community pharmacy professionals (CPPs) in preventing and managing noncommunicable diseases in Ethiopia remains inconsistent. This study aimed to synthesize available research on the roles and barriers faced by CPPs in noncommunicable diseases prevention and management among adults in Ethiopia.

**Methods:**

A comprehensive literature search using four electronic databases, including Scopus, Medline/Ovid, Web of Science, and Embase was conducted. In addition, further studies were identified through Google Scholar searches and manual reference searches. The review included studies published before 30 July 2024 without any limitations on the starting time.

**Results:**

A total of 15 studies were included in this review, with most studies focused on CPPs’ involvement in the prevention and management of non-specific NCDs and diabetes. CPPs were involved in a variety of roles in the prevention, screening, and management of NCDs. Their scope of practice included providing health promotion on lifestyle changes and nutrition, medication therapy management, chronic disease screening, disease-specific counseling, and general self-care promotion. This review identified different barriers CPPs encountered during their provision of NCD care, which were mainly categorized under four classes: CPP-related, pharmacy setting and working environment-related, policy/healthcare system-related, and patient/public-related barriers.

**Conclusion:**

CPPs provide a wide range of services, from health promotion to disease screening and management of noncommunicable diseases. However, potential barriers such as limited training and resources, lack of reimbursement for noncommunicable disease services, and inadequate integration into the healthcare system can limit them from effectively providing these services. Addressing these barriers is essential to enhance the role of CPPs in noncommunicable diseases prevention and management.

**Systematic review registration:**

https://www.crd.york.ac.uk/PROSPERO/view/CRD42023486384, Unique Identifier: CRD42023486384.

## Introduction

Chronic diseases are major public health challenges worldwide, accounting for a significant portion of morbidity and mortality. According to the World Health Organization (WHO), noncommunicable diseases (NCDs), including heart disease, stroke, cancer, diabetes, and chronic lung disease, are collectively responsible for 74% of all deaths worldwide. Over 75% of all NCD deaths, and 86% of the 17 million people who died before the age of 70, occur in low- and middle-income countries (1).

Ethiopia has made significant progress on social development, including poverty reduction, with encouraging results in communicable disease control, improved nutrition, and maternal and child health (2). However, the country is also experiencing an epidemiological shift, with a rapidly increasing prevalence of NCDs, which calls for new and innovative public health strategies to address the double burden of diseases. Studies conducted by the Federal Ministry of Health in 2015 (STEPS Survey) showed evidence of an increasing burden of NCDs and their risk factors, driven by factors such as population aging, urbanization, and lifestyle changes (3). The four most common NCDs in Ethiopia are cardiovascular diseases, diabetes, cancer, and chronic respiratory diseases, which account for a significant portion of the country’s healthcare burden, both in terms of cost and lost productivity (4).

The Ethiopian healthcare system faces numerous challenges in preventing and managing NCDs (5). These challenges include a shortage of healthcare workers, particularly specialists in chronic disease management, limited access to essential medications and diagnostics, low patient awareness of NCDs and their risk factors, and lack of adherence to treatment regimens (6, 7). These factors hinder effective prevention and management, leaving many Ethiopians undiagnosed, untreated, or with inadequate care (8–10).

The International Pharmaceutical Federation reveals that pharmacists are increasingly being recognized as valuable members of the healthcare team in the prevention and management of NCDs (11). Pharmacists are readily accessible to patients, have a strong understanding of medications, and can provide individualized counseling and support to patients with different medical conditions (12–21). Studies worldwide have demonstrated that community pharmacists can effectively deliver chronic disease prevention and management services, including medication therapy management, disease monitoring, and patient education (15, 22–29).

In Ethiopia, patients with noncommunicable diseases are typically diagnosed and initiated on treatment by clinicians at the primary healthcare level (30). Once their condition stabilizes, they may obtain medication refills at community pharmacies using valid prescriptions. This shift is largely driven by the higher cost and limited accessibility of services, such as physician consultations, laboratory services, and medicines, within the primary healthcare facilities. Convenience and accessibility are also the main reasons that patients choose community pharmacies.

Community pharmacy professionals (CPPs) have increasingly contributed to the prevention and management of noncommunicable diseases (31–35). Beyond dispensing medications, pharmacists provide minor illness management, lifestyle counseling, and health education. Their strong understanding of pharmacotherapy management enables them to provide effective medication counseling, monitor adherence and adverse effects, and refer patients to other healthcare providers when necessary (22, 34). Given the wide availability of pharmacies across the country, CPPs are well-position to support chronic disease care and improve access to prevention and management services (36).

A preliminary review of existing studies on CPPs’ roles in noncommunicable disease prevention and management in Ethiopia revealed a lack of generalizability and inconsistent findings due to varying study designs, methodologies, and outcomes. This gap highlights the need for a comprehensive study that assesses the scope of practices and barriers to effective engagement in preventing and managing noncommunicable diseases among CPPS. Therefore, this study aimed to comprehensively synthesize existing evidence regarding CPPs’ roles and perceived barriers in the prevention and management of noncommunicable diseases in Ethiopia. The review first discusses CPPs’ scopes of practice and then follows a presentation of the barriers hindering their full engagement in the prevention and management of noncommunicable diseases.

## Methods

### Registration and reporting

The protocol for this review was registered in the PROSPERO database (CRD42023486384) and is publicly accessible. The protocol has been revised after observing the works of literature in the initial search outcome. Modifications were made and expanded regarding inclusion and exclusion criteria. The Preferred Reporting Items for Systematic Reviews and Meta-Analyses (PRISMA) 2020 checklist was used to guide the reporting of this systematic review (37) (Supplementary file S1).

### Eligibility criteria

We included eligible studies that examined the roles and/or barriers of CPPs in preventing and managing NCDs in adults within community settings. Studies that addressed both roles and barriers, or either one, were included based on the following inclusion and exclusion criteria.

#### Inclusion criteria

Articles that met the following criteria were considered for inclusion in this systematic review.

- Study population: CPPs [pharmacists with bachelor’s degree holders and above and diploma holders (pharmacy technicians)].
- Intervention (counseling, screening, health promotion and education, and medication management): studies reported about potential roles CPPs played in counseling, screening, medication management, and health promotion and education in the prevention and/or management of NCDs and/or barriers in providing these practices.
- Outcome (preventing and managing NCDs): studies conducted on one or more of these NCDs, including cardiovascular diseases, diabetes, chronic respiratory diseases, and cancer in adults.
- Context: Ethiopia.
- Study design: no restriction.
- Study setting: community drug retail outlets (CDROs), including pharmacies and drug stores located in Ethiopia.
- Population served by CPPs: adults (age ≥ 18 years).
- Time: no restriction for starting and articles published until July 30, 2024.
- Language: studies conducted in English.
- Type of studies: limited to published peer-reviewed primary studies and thesis and/or dissertations were included in this study.

#### Noncommunicable chronic diseases

This review determined chronic disease based on the WHO definition. Chronic diseases are not contagious and typically last a long time, often progressing slowly (38). Our review also ensured the inclusion of articles that involved these main types of NCDs, such as cardiovascular diseases (including hypertension, heart attacks, and stroke), cancers, chronic respiratory diseases (such as chronic obstructive pulmonary disease and asthma), and diabetes. Although metabolic syndrome is not classified among the four main categories of NCDs, it was included in our review due to its association with cardiovascular and high blood glucose issues, and its role as a major risk factor for other NCDs.

Therefore, this review included all articles focused on the roles and barriers of CPPs in the management and prevention of one or more specific or non-specified NCDs in adults.

#### Exclusion criteria

Research articles such as reviews, letters to editors, short communications, commentaries, book chapters, and conference abstracts were excluded from this review.

### Data sources

A comprehensive literature search was conducted, with an initial database search on 15 January 2024 and finalized on 30 July 2024, using four electronic databases: Scopus, Medline/Ovid, Web of Science, and Ovid Embase. In addition, further studies were identified through a manual Google Scholar search and manual reference searching of included studies. This study included all studies published in Ethiopia before 30 July 2024 without any other limitations.

### Search strategy

**Figure 1:**
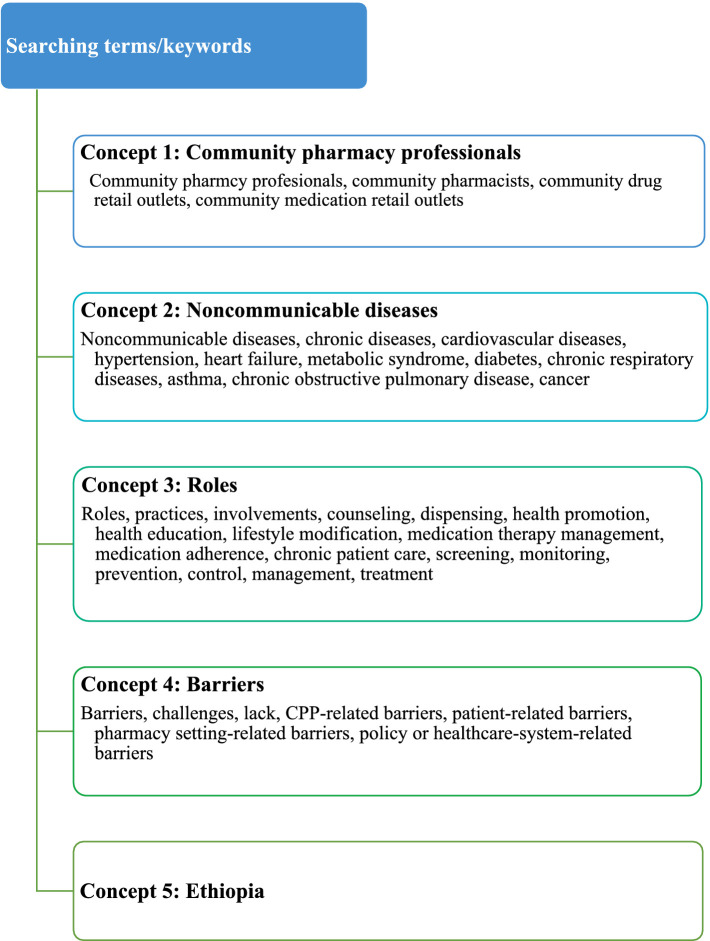
Summary of search terms/keywords used in articles searching for each concept.

The search strategy aimed to identify studies reporting on the roles and barriers of CPPs in the prevention and management of NCDs in Ethiopia. Search terms related to CPPs, NCDs, chronic diseases, roles, and barriers were combined using Boolean operators “OR” and “AND” to develop the search strategy (Figure 1). The comprehensive search strategies used in the Medline and Scopus searches are available in the Supplementary file S2.

### Study selection process

Identified records from individual databases were exported to EndNote version 20 and then to Covidence for screening. After de-duplication, eligible records were screened starting from 30 July 2024 using title and abstract for full-text retrieval by the first investigator (AKS). Finally, a full-text screening was carried out by three investigators (AKS, EAB, and LWL) independently to identify eligible articles using predetermined inclusion and exclusion methods. Disagreements were resolved through discussion using specified criteria.

### Data extraction process

After identifying full-text screened studies, data were extracted by three investigators (GSC, ADD, YBT) using a tailored data extraction format prepared by the research team in Microsoft Excel. Data extracted included the name of the first author, publication year, study design, total number of participants, level of pharmacy professionals (bachelor’s and above and diploma), types of NCDs, reported roles in preventing and managing NCDs, and reported barriers of CPPs in preventing and managing NCDs. Disagreements between authors that arose during screening and data extraction were resolved through discussion using specified criteria and the involvement of other investigators (KYG, FBT, BBA).

### Quality assessment

The quality of the included articles was assessed using the Joanna Briggs Institute (JBI) quality appraisal tools for analytical cross-sectional studies (39). Three investigators (AKS, GKB, BBA) independently assessed the quality of the included articles using the JBI critical appraisal checklist, which consists of eight criteria: (1) clear inclusion and exclusion criteria; (2) description of the study subject and setting; (3) use of a valid and reliable method to measure exposure; (4) standard criteria used for measurement of the condition; (5) identification of potential confounding factors; (6) development of strategies to deal with confounding factors; (7) use of a valid and reliable method to measure the outcomes; and (8) use of appropriate statistical analysis. To determine the risk of bias, studies were classified into three categories based on their total scores: Low risk (score of 6 to 8), moderate risk (score of 3 to 5), and high risk (score of 0 to 2). Only studies with low or moderate risk of bias were included in this review. Disagreements that arose during the full-text quality assessment were resolved through evidence-based discussion with the involvement of other review authors (AKS, LWL, FBT).

### Data synthesis

The data for both roles and barriers were thematized for synthesis and presentation. Thematic areas in terms of types of NCDs: (I) NCDs (non-specified), (II) diabetes, (III) cardiovascular diseases, (IV) asthma, and (V) metabolic syndrome. The roles of CPPs were categorized under: (I) general health education and promotion services, (II) promotion related to nutrition and lifestyle modification, (III) medication therapy management, (IV) chronic disease screening and counseling, and (V) disease-specific and other counseling. Barriers were categorized under four themes based on the sources and nature of the barriers: (I) CPP-related, (II) pharmacy setting and work environment-related, (III) policy and healthcare system-related, and (IV) patient/client or public-related barriers.

## Results

### Screening results

**Figure 2:**
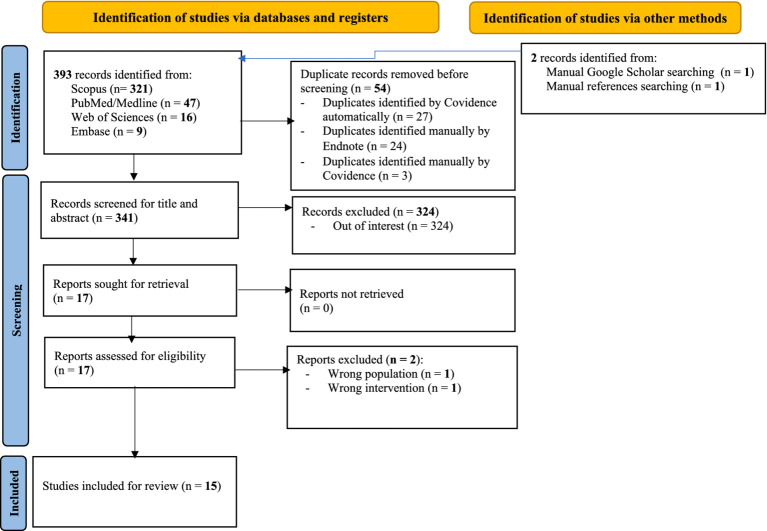
PRISMA flow diagram of included articles.

A total of 393 articles were identified using electronic databases and two manual searches of other relevant sources. After removing duplicate records, 341 records were screened. Following the exclusion of 324 articles based on titles and abstracts, 17 were assessed in full text. Two articles were excluded (one for not addressing the phenomena of interest and one for including a non-target population), resulting in 15 studies included in the review. The PRISMA flow diagram summarizes the selection process (Figure 2).

### Characteristics of included studies

The included studies were published between 2017 and 2024. Except for one non-peer-reviewed master’s thesis study found in the repository of Addis Ababa University (40), all were peer-reviewed articles. Most studies were conducted in Gondar City and nearby (22, 34, 41–44) and Amhara region using multi-center study designs (31–33, 45–47). Two studies were carried out in Addis Ababa City (40, 48), and one study in Injibara City (49). Most of the studies were conducted using a cross-sectional survey design, except two studies used cross-sectional plus simulated patient (SP)-based scenarios to observe actual practices (31, 42). The reviewed studies included several participants ranging from 24 (49) up to 412 (46, 47) (Table 1).

**Table 1:** Characteristics of included articles related to roles and barriers of community pharmacy professionals in the prevention and management of noncommunicable diseases in Ethiopia.

S. no	Authors	Publication year	Study area	Study design	Number of participants	Bachelor’s degree and above	Diploma holders	Main outcome	Study’s strengths and limitations
1	Belachew et al. (41)	2024	Gondar City	Cross-sectional	100	48	52	Most CPPs had a high knowledge of therapeutic nutrition. They had a positive attitude toward nutritional assessment. Key barriers were low patient awareness and demand for nutrition counseling.	Limited generalizability due to single-centered and small sample size. It used purposive sampling.
2	Sendekie et al. (31)	2024	Multi-center (Northwest Ethiopia-3 cities)	Cross-sectional & SP	184- Survey, 245-SP	42	58	Most CPPs had low involvement in the actual practice of managing and screening patients with diabetes.	Provides valuable insights into CPPs’ role in diabetes management. The study was limited to urban settings.
3	Sendekie et al. (32)	2023	Multi-center (Northwest Ethiopia-3 cities)	Cross-sectional	184	77	107	Most CPPs perceived to have high involvement in diabetes management. However, education, income, and working hours influenced their involvement.	The study did not address rural settings.
4	Sendekie et al. (33)	2023	Gondar City	Cross-sectional and SP	71-survey, 213-SP	44	27	CPPs were highly involved in dispensing prescription-only cardiovascular medications without a prescription and did not provide proper patient counselling, education, and screening.	Limited generalizability due to small sample size.
5	Sendekie et al. (42)	2023	Multi-center (Northwest Ethiopia-3 cities)	Cross-sectional	285	161	124	Most CPPs were actively engaged in promotional activities in preventing and managing NCDs. Barriers include inadequate counseling areas and poor coordination with other healthcare providers.	It provides valuable insights into CPPs’ involvement in NCD health promotion, but limited generalizability, did not account for rural areas, and potential biases in self-reported data.
6	Ayenew et al. (49)	2022	Injibara City	Cross-sectional	24	9	15	CPPs were willing and positive toward health promotion services, but barriers included lack of knowledge, time, confidence, training, space, and management support, as well as employer reluctance to expand their scope, regulatory restrictions, and absence of standard guidelines.	It guides further research on CPPs’ involvement and informs strategies to improve their participation in healthcare. However, the small sample size and focus on one city limit the generalizability of the results.
7	Birarra et al. (43)	2022	Gondar City	Cross-sectional	81	36	45	Most CPPs were knowledgeable about cardiovascular and NCDs management and prevention, but most did not routinely measure blood pressure in hypertensive clients.	A small sample size, cross-sectional study has a low cause-and-effect relationship.
8	Sendekie et al. (22)	2022	Multi-center (Gondar City and nearby rural towns)	Cross-sectional	210	41	169	CPPs provided counseling on cardiovascular disease prevention and management and had a good understanding of strategies. However, their involvement in measuring weight, blood pressure, and glucose levels, dispensing monitoring equipment, and maintaining patient records is limited.	Small sample size and the self-reported nature of the response may affect outcomes due to social desirability bias.
9	Emiru et al. (45)	2020	Multi-center (Amhar region-5 cities)	Cross-sectional	122	55	67	Most pharmacists believed they could identify and manage asthma triggers and provide basic information about the disease and medications. However, they reported it needed more time and a suitable environment to effectively counsel patients on disease management and medication.	The cross-sectional study relied on self-reported practices, which may not accurately reflect actual practices due to social desirability bias.
10	Belachew et al. (34)	2020	Gondar City	Cross-sectional	65	45	20	The overall involvement of CCPs in counseling patients, opinion about metabolic syndrome, and perception toward the effectiveness of the intervention was found to be positive. However, the provision of services, such as monitoring therapy, selling equipment for home blood pressure and glucose monitoring and documenting patient care services needs to be encouraged.	It fills a gap in community pharmacy practice literature in Ethiopia, with a high response rate and a clear, structured questionnaire. Its limitations include its cross-sectional nature, potential respondent bias, and reliance on self-reported data and recall.
11	Moges (40)	2019	Addis Ababa City	Cross-sectional	297	214	83	Most CPPs were willing to perform health promotion functions, with strengths in promoting physical activity, screening for diabetes, and healthy eating. However, they feel less equipped to use test kits for cholesterol screening or explain the harms of khat chewing. Barriers include the lack of guidelines, insufficient space for privacy, and knowledge and skill gaps, leading to low involvement in health promotion.	The questionnaire for quantitative data was validated. Utilized mixed methods. Limitations include recall bias, respondent error, incomplete surveys, and the study’s focus on an urban setting, limiting generalization to rural communities.
12	Teka et al. (48)	2018	Addis Ababa City	Cross-sectional	300	221	79	CPPs provided proper counseling on the timing of oral anti-diabetic drugs and missed doses, but very small gave proper counseling on the importance of continuous screening for nephropathy, retinopathy, and neuropathy.	Use a self-administered questionnaire to mitigate social desirability bias. A cross-sectional study has a low cause-and-effect relationship.
13	Erku et al. (46)	2017	Multi-center (Amhar region-6 cities)	Cross-sectional	412	333	79	CPPs had poor knowledge and low involvement in counseling and health education for diabetes patients, with lack of knowledge and clinical skills as the main barriers	Limitations include being a cross-sectional study conducted in only six cities in Amhara region. The use of self-administered questionnaires may introduce social desirability bias.
14	Asmelashe et al. (44)	2017	Gondar City	Cross-sectional	48	27	21	Counseling on drug misuse and asthma was most common, while traditional medicine and cancer counseling were least performed. Service quality was rated good, with satisfaction reported. The main barrier was a lack of training, and involvement in health promotion varied by sex, education, and pharmacy ownership.	Limited scope in scope and quality of health promotion services. Social desirability bias due to the self-administered questionnaire.
15	Erku et al. (47)	2017	Multi-center (Amhar region-6 cities)	Cross-sectional	412	233	179	The survey revealed low involvement of community pharmacy professionals in public health services, particularly in lifestyle counseling and screening. The main barrier was a lack of knowledge or clinical skills.	The findings may not be generalizable to other regions. The self-administered questionnaire could introduce respondents or recall bias.

SP, simulated patient.

### Quality assessment

The quality assessment results, conducted using the JBI quality appraisal tools for analytical cross-sectional studies, showed that all included articles scored 6–7 out of 8 and were found to be in the range of low risk of bias (22, 31–34, 40–49) (Supplementary file S3).

### Types of NCDs managed by community pharmacy professionals

Among the included studies in this review, six studies explored the involvement of CPPs in non-specific NCD prevention and management practices (33, 40, 41, 44, 47, 49) and addressing the most common chronic diseases such as diabetes, cardiovascular diseases, asthma, and cancer. Four studies were conducted about the involvement of CPPs in the management of patients with diabetes (31, 32, 46, 48), and three studies were conducted focusing on CPPs’ involvement in managing cardiovascular diseases (22, 42, 43). The remaining two studies assessed CPPs’ practices regarding asthma (45) and metabolic syndrome (34) (Table 2).

**Table 2:** Studies explored community pharmacy professionals’ involvement regarding types of noncommunicable diseases in the prevention and management in Ethiopia.

S. no	Types of NCDs pharmacists involved	Number of included studies	Number of included pharmacy professionals
1	Non-specific NCDs	6	1,166
2	Diabetes	4	1,080
3	Cardiovascular diseases	3	362
4	Chronic respiratory diseases (Asthma)	1	122
5	Metabolic syndrome	1	65

NCDs, noncommunicable diseases.

### Roles of community pharmacy professionals in preventing and managing NCDs

Studies revealed that CPPs were involved in various health education services, including health education on screening, risk factors, and the burden of NCDs (complications and economic and social problems) (22, 32–34, 44), primary health prevention activities related to counseling on nutrition and lifestyle changes (22, 31–34, 40–44, 46–49), and medication therapy management counseling (22, 31–34, 45, 46, 48). Additionally, despite limitations in terms of the number of CPPs involved, CPPs were involved in the screening of chronic diseases such as hypertension and diabetes (22, 31–34, 40, 43, 47), asthma (45, 47), cholesterol screening (40, 43, 47), and cancer screening (44). Although the number of CPPs involved varied across studies, disease-specific and other counseling practices, such as counseling on retinopathy, nephropathy, neuropathy screening, foot care, and eye care for patients with diabetes (40, 46), counseling patients to consult clinicians/physicians (22, 31–34), and immunization counseling related to certain NCDs, including cancer, for an adult population were within the scope of CPPs’ practices (44, 49) (Table 3).

**Table 3:** Scopes of practice and specific activities of community pharmacy professionals involved in the prevention and management of noncommunicable diseases in Ethiopia.

Category of CPPs’ scopes of practice	Specific and detailed roles of CPPs
General health education services	Health education on screening of NCDs
	Health education related to risk factors of NCDs
	Health education about the burdens of NCDs (complications, economic, and social problems)
Primary prevention counseling related to nutrition and lifestyle changes	Health education on nutrition diet and therapy
	Health education on low cholesterol-containing diets and vegetables
	Health education on smoking cessation
	Health education related to physical activity
	Health education related to salt restriction
	Health education on risky alcohol restriction
	Health education related to weight management
Medication therapy management	Counseling on medication adherence
	Dosage regimen counseling
	Therapeutic response monitoring
	Adverse effect monitoring
	Prescription-only-medications counseling
	Caution of over-the-counter drugs and herbal products
	Counseling on medication storage conditions and handling practices
Secondary prevention activities (Screening and counseling on screening)	Weight measuring
	Hypertension
	Diabetes (blood glucose)
	Asthma screening
	Cholesterol
	Cancer screening counseling
Disease-specific and other counseling	Counseling on retinopathy, nephropathy, and neuropathy screening for diabetes patients
	Counsel to foot care and eye care for patients with diabetes
	Counsel patients to consult clinicians/physicians
	Immunization counseling

CPPS, community pharmacy professionals; NCDs, noncommunicable diseases.

### Barriers to community pharmacy professionals in preventing and managing NCDs

Of the 15 studies included in this review, 10 studies reported potential barriers that influence CPPs’ active involvement in preventing and managing NCDs. The identified barriers were mainly related to CPP-related factors, pharmacy setting and working environment factors, policy and healthcare system factors, and patient/client and public factors (22, 33, 40, 41, 43–47, 49). The most reported high levels of barriers were workload/lack of time, lack of clinical knowledge and skills, lack of resources such as updated and standardized guidelines, limited on-the-job training and continuing education, and lack of appropriate and standardized private counseling areas, and lack of follow-up and service monitoring from regulatory bodies (Table 4).

**Table 4:** Barriers to the involvement of community pharmacy professionals in noncommunicable disease prevention and management in Ethiopia.

Category of reported barriers	Specific and detailed activities of community pharmacy professionals
CPPs-related barriers	Workload/lack of time
	Lack of clinical knowledge and skills
	Unable to update themselves and lack updated knowledge
	Limited on-the-job training and continuing education
	Lack of communication with other healthcare providers
	Lack of access to patient medical records
Pharmacy setting and working environment-related barriers	Lack of appropriate and standardized private counseling areas in CDROs
	Limited number of CPPs in the pharmacy
	Lack of support from managers
	Lack of resources, such as updated and standardized guidelines in the pharmacy
	Unwilling to pay for wider scopes of practice from owners
	Poor collaboration with other healthcare settings
Policy and healthcare system-related barriers	Lack of follow-up and service monitoring from regulatory bodies
	Low provision of on-the-job training for CPPs
	Lack of provision of standardized and customized guidelines and frameworks
	Lack of financial and remuneration frameworks for supporting service providers
Patient/client and public-related barriers	Limited awareness of the public/clients about CPPs’ provided service
	Lack of demand from patients/clients to receive services

CPPS, community pharmacy professionals; CDROs, Community drug retail outlets.

## Discussion

NCDs pose a significant global health challenge, and CPPs are increasingly recognized as key players in their prevention and management. This review highlights the role of Ethiopian CPPs in NCD care, revealing a range of activities from health promotion to disease screening and counseling. However, different barriers that need to be addressed are also identified in this review.

### Community pharmacy professionals’ roles in the prevention and management of NCDs

To tackle the increasing burden of chronic diseases, the involvement of stakeholders is crucial, and incorporating CPPs in this role could be instrumental. The findings from this review revealed that CPPs are involved in general health promotion, nutrition, lifestyle education, and medication therapy management. They also engage in screening for chronic diseases such as hypertension, diabetes, asthma, and cholesterol. Furthermore, CPPs provide disease-specific counseling services, demonstrating a level of care that is often lacking in primary healthcare settings. However, inconsistency in the scope of CPPs’ practices across different studies raises concerns about the accessibility and availability of these services. Further research is warranted to understand the factors influencing the variation in CPPs’ involvement across different regions of Ethiopia.

This review highlights the potential of CPPs in preventing and managing NCDs. The current findings align with evidence from reviews in low- and middle-income countries, which has shown a positive impact of community pharmacies in NCD services (50–53). Another primary study in low- and middle-income countries, such as Nepal, has shown a positive impact of the community pharmacy workforce in preventing and managing NCDs (54). Studies from Lesotho (55), Ghana (25, 56) and Nigeria (23, 24, 57) report similar involvement in health promotion, medication therapy management, and disease screening. A study in Kuwait also revealed a significant role of CPPs in preventing and managing metabolic syndrome (26). These findings have the potential to expand the role of the pharmacy workforce in primary healthcare globally. However, the extent of these activities varies significantly across countries, influenced by regulatory frameworks, healthcare system structures, and level of economic development.

There is a growing trend worldwide toward expanding the role of CPPs in NCD care (28, 58). Countries such as the United Kingdom (59), Australia (60, 61), and Canada (62–64) have implemented pharmacist-led models that include disease management programs, medication reviews, and chronic disease screening. Evidence shows a significant impact of pharmacists on diabetes management and screening (65, 66). In countries with well-established healthcare systems, community pharmacists play a vital role in NCD prevention and management, with an umbrella review demonstrating their contributions to improved clinical outcomes in diabetes, hyperlipidemia, cardiovascular diseases, and respiratory conditions (67). However, findings from this review reveal that CPPs’ involvement in Ethiopia varies based on their educational background. Many practitioners are diploma-level graduates or did not receive regular on-the-job training, influencing their capacity for service delivery. This underscores the need for training and continuing education to enhance their effectiveness.

The findings from this review indicate a strong focus on health promotion and education related to nutrition and lifestyle changes, which are crucial for NCD prevention. This aligns with global recommendations for pharmacists’ role in promoting healthy behaviors (68). Other African countries have reported similar efforts in NCD prevention, with pharmacists providing counseling on diet, exercise, and smoking cessation (50, 69). A systematic review also highlights community pharmacists’ significant role in preventing cardiovascular disease risk factors through health promotion and education related to nutrition and lifestyle changes (70). However, the intensity and reach of pharmacists’ roles vary considerably. For instance, in developed countries, pharmacists are actively involved in vaccination programs, such as HPV and hepatitis B, which, while targeting infectious agents, contribute to the prevention of certain NCDs like cervical and liver cancers (71). While this aspect is not explicitly covered in the studies reviewed here, it is a critical component of pharmacists’ health promotion role in other regions.

This review also highlights the involvement of CPPs in screening for hypertension, diabetes, asthma, and cholesterol, which is commendable given the resource constraints. Screening for NCDs is gaining momentum in many African countries, with pharmacists playing an increasingly important role (72–74). However, access to diagnostic tools and trained personnel remain a challenge. A study in Saudi Arabia reveals pharmacists’ roles in cardiovascular disease screening (75), while studies in Brazil, India, South Africa, and the United States (76), Australia (66) showed a significant engagement of pharmacists in chronic disease screening. However, findings from this review indicate that screening practices are limited and vary across settings, highlighting the need for further evaluation of the sustainability of these services and their impact on patient outcomes.

Notably, none of the included studies in this review explicitly address the involvement of CPPs in cancer screening, except for one study that reported CPPs providing cancer screening counseling (44). However, findings from other systematic reviews (77) and studies in Canada (78) suggest that community pharmacists can contribute to cancer screening. The current findings indicate the need to empower CPPs to play a greater role in cancer screening, thereby increasing their public health impact.

This review also highlights CPPs’ role in medication therapy management for patients with NCDs. In many developed countries, pharmacists are at the forefront of NCD management, providing comprehensive medication reviews, adherence counseling, and self-management support (79–81). They work closely with patients, physicians, and other healthcare professionals to optimize treatment outcomes. While medication therapy management is recognized as a crucial role for CPPs in NCD management, findings from this study indicate wide variations in implementation across studies, largely influenced by CPPs’ educational backgrounds and training levels. These disparities underscore the need for further research and policy reforms to strengthen and standardize the role of CPPs in NCD prevention and management in developing countries.

### Barriers to community pharmacy professionals in the prevention and management of NCDs

The review’s findings underscore the significant challenges CPPs face in Ethiopia in their efforts to contribute to chronic disease prevention and management. Identified barriers were categorized into four key barrier categories—CPPs-related, pharmacy setting and working environment-related, policy and healthcare system-related, and patient/client and public-related barriers.

The review identified CPPs-related barriers such as lack of knowledge, skills, workload, lack of resources, limited training and continuing education, and lack of collaboration with other healthcare providers as potential barriers. These challenges are not unique to Ethiopia and are commonly reported in other African countries (51, 69). However, the extent of these barriers may vary depending on the level of pharmaceutical education and training available.

This review discloses that CPPs in Ethiopia lack adequate training and resources to effectively manage chronic diseases. Most studies indicated that a significant number of CPPs had attained a diploma-level education. They may not have access to up-to-date clinical guidelines, diagnostic tools, or patient education materials (22, 33). Globally, there is a growing recognition of the need for continuous professional development for pharmacists to equip them with the necessary competencies to manage chronic diseases (82). Many countries have implemented structured training programs and certification schemes to address this gap (83). To improve the public health services provided in community pharmacies, training must aim to increase CPPs’ confidence in providing these services.

Workload and time constraints are also mentioned as main sources of barriers. CPPs face multiple demands in their roles, including dispensing medications, managing administrative tasks, responding to urgent patient needs, and providing counseling and clinical services. These pressures often limit their capacity to deliver optimal patient care. This finding is in line with findings from Senegal (84) and Australia (85). The finding suggests that assigning enough CPPs can help provide optimal service provisions.

As highlighted in the review, the pharmacy setting and working environment in Ethiopia present significant challenges, such as inadequate counseling areas and resources, a limited number of CPPs and a lower workforce, lack of support from managers, and inadequate integration of pharmacies with other healthcare settings. These conditions are common in many African countries due to limited healthcare infrastructure and economic constraints (51, 86). Globally, there is a wide variation in pharmacy settings (87). While some countries have relatively advanced pharmacy systems, many others, particularly in parts of sub-Saharan Africa, face similar challenges to those in Ethiopia, where the healthcare system struggles with limited resources, inadequate staffing, and infrastructure issues (88). However, the impact of these barriers on CPPs’ ability to provide optimal care for patients with chronic diseases can be mitigated through innovative service delivery models and the use of technology (89, 90).

The review identified healthcare system-related barriers such as a lack of follow-up and service monitoring from regulatory bodies, a lack of standardized and customized guidelines and frameworks, a lack of financial and remuneration frameworks for supporting service providers, and a lack of integration frameworks of pharmacies into the healthcare system. These challenges are prevalent in many low- and middle-income countries, including those in Africa (51, 86). Globally, healthcare systems vary widely in terms of their organization, financing, and delivery (91). A communication and collaboration framework between CPPs and other healthcare providers should be instrumental in facilitating the integration of CPPs into chronic disease management teams (92). In addition, CPPs engaging in clinical practice in Ethiopia often receive inadequate remuneration, which can lead to recruitment and retention challenges (93). Furthermore, their professional contributions may not be fully recognized or valued within the healthcare system. The policy and regulatory framework governing community pharmacy practice in Ethiopia may not fully support their involvement in chronic disease management. Clear guidelines and protocols are needed to define their role, responsibilities, and scope of practice.

Patient and/or public-related barriers, such as limited awareness of the services provided by CPPs and lack of demand from patients/clients to receive these services, have been identified in the review. These challenges are common across different populations and cultural contexts. Public-related barriers, such as negative attitudes toward chronic diseases and a lack of awareness of the role of CPPs, may also have been highlighted (94). Addressing these barriers requires comprehensive public health campaigns and educational initiatives to raise awareness of the benefits of CPPs’ services and to promote positive attitudes toward chronic disease management.

In general, beyond the barriers discussed in this review, patients with NCDs have multiple healthcare needs and multiple medications, usually because of comorbidities and complications. Access to healthcare services, including the availability of physicians, consistent supply of essential medicines and affordability, and broader socioeconomic factors, can significantly influence the prevention and management of NCDs in Ethiopia, as well as in other resource-limited countries across Africa (95). These systemic and structural challenges may lead patients to seek alternative sources of care other than primary healthcare levels, such as community pharmacies. To leverage the accessibility of community pharmacies in managing NCDs in Ethiopia and similar settings, a strategic approach is needed. This includes formally integrating pharmacies into the primary healthcare system and expanding pharmacists’ roles to include counselling, screening, and referrals. Training and clear clinical guidelines should support this shift. Ensuring consistent access to affordable medicines through strengthened supply chains is essential. Digital linkages with primary healthcare facilities can improve care coordination, while pharmacies can also serve as key platforms for raising health awareness and promoting treatment adherence.

### Strengths and limitations of this study

This study is the first of its kind and presents comprehensive findings regarding CPPs’ roles and barriers in preventing and managing NCDs in Ethiopia. These findings can be used to enhance the practice of CPPs and as an instrument to update policies and frameworks to incorporate CPPs as potential stakeholders in addressing the burden of NCDs. However, this review has some limitations that should be considered when interpreting the findings. The included studies were not evenly distributed across the country’s regions, with a particular concentration in the Amhara region and Addis Ababa, primarily focusing on urban settings. Additionally, the included studies addressed the role of CPPs in limited types of NCDs, which may limit the generalizability of the findings. Furthermore, the review did not differentiate between pharmacists and diploma-level pharmacy technicians in the analysis, although their roles and training differ significantly. This lack of distinction may affect the interpretation of both roles and barriers when compared to similar studies that focused exclusively on pharmacists. Finally, this review could not address the level and proportion of CPPs’ involvement in each scope of practice, and the magnitude of the barriers CPPs face.

### Implication of findings

Implications for policymakers: This review can inform the development of evidence-based guidelines and policies that empower CPPs to effectively contribute to NCD prevention and management in the Ethiopian context. Recognizing the potential of CPPs in NCD care, policymakers should develop policies and strategies to:

- Integrate CPPs into the broader healthcare system.
- Strengthening the role of CPPs by allocating adequate resources and investing in infrastructure.
- Develop and implement comprehensive training programs to enhance CPPs’ knowledge and skills in NCD prevention, screening, and management.
- Establish and implement appropriate reimbursement mechanisms for CPP-provided NCD services.
- Foster collaboration between CPPs, physicians, and other healthcare providers.

Implications for CPPs: CPPs should actively engage in continuing education to enhance their knowledge and skills in NCD management. They should also advocate for policies that support their expanded role in NCD care. Building strong collaboration with other healthcare providers can improve patient outcomes and enhance CPP’s role. CPPs can also explore innovative service delivery models to address the challenges of NCD care in resource-limited settings, such as telepharmacy or point-of-care testing.

Implications for research: Further research is needed to identify effective interventions to address CPPs’ barriers in Ethiopia’s NCD care. Studies should evaluate the impact of CPP-led NCD care programs on patient outcomes, healthcare costs, and patient satisfaction. Exploring patient perspectives on CPP-provided NCD services would be important to inform service improvement and ensure these services align with patient needs.

### Recommendations to tackle potential barriers

To address barriers hindering CPPs in NCD prevention and management in Ethiopia, targeted interventions are needed.

CPPs-related barriers can be mitigated through structured continuous professional development programs, on-the-job training, and access to updated guidelines and digital health tools. Strengthening interprofessional collaboration and expanding access to electronic medical records will enhance service delivery.

Pharmacy setting barriers require investment in private counseling areas, workforce expansion, and regulatory enforcement of standardized guidelines. Strengthening managerial support and fostering collaboration with healthcare institutions will improve working conditions and service integration.

Policy and healthcare system barriers can be addressed by enhancing regulatory oversight, routine follow-ups, and sustainable financing models, including reimbursement schemes. Standardized, Ethiopia-specific guidelines should be developed to ensure clarity in CPPs’ roles.

Patient and public-related barriers require awareness campaigns through media and community engagement to highlight CPPs’ contributions to NCD care. Integrating CPPs into primary healthcare teams can enhance public trust and service demand.

By implementing these targeted strategies, Ethiopia can optimize the role of CPPs in reducing the NCD burden and improving public health.

## Conclusion

The findings from this review highlight the significant potential of CPPs in supporting the prevention and management of NCDs in resource-limited settings such as Ethiopia. However, as most of the current studies are from the Amhara region and Addis Ababa, the findings may not reflect pharmacy practices in other Ethiopian regions and remote areas. Although CPPs are already engaged in NCD prevention, screening, and management, there remains considerable room for improvement in terms of service consistency, accessibility, and integration within the broader healthcare system. By addressing these challenges and drawing lessons from the experiences of other African countries and global best practices, Ethiopia can enhance the role of CPPs in NCD care. Key strategies include investing in CPP training, ensuring the provision of adequate resources, and developing clear guidelines and protocols for delivering NCD-related services.

## Data Availability

The original contributions presented in the study are included in the article/Supplementary material, further inquiries can be directed to the corresponding author.
