# Supplementary material for: Roles and barriers of community pharmacy professionals in the prevention and management of noncommunicable diseases in Ethiopia: a systematic review
Source: Front Public Health. 2025 Aug 28;13:1485327. doi: 10.3389/fpubh.2025.1485327 (PMC12422921; doi:10.3389/fpubh.2025.1485327)
Supplement: Supplementary file 2 [file Data_Sheet_2.pdf]

No	Keywords	Searching term
1	Community pharmacists	Pharm* OR "Communit* pharm*" OR "communit* pharm* profession*" OR "communit* drug retail*" OR "drug retail*" OR "communit* medic* retail*" OR CPPs
2	Noncommunicable diseases	"non?commun* diseases" OR "chronic* disease*" OR "non?communicable*ADJ3 disease*" OR "cardiovascular disease*" OR hypertension OR "heart failure" OR "metabolic syndrome" OR diabetes OR "chronic* respiratory disease*" OR asthma OR "chronic* obstructive pulmonary disease*" OR cancer OR NCDs
3	Roles	role* OR practices OR involvements OR counseling OR dispensing OR "health promotion*" OR "health education" OR "lifestyle modification" OR "medic* therapy management" OR "medic* adherence" OR "chronic* patient* care*" OR screen* OR monitor* OR prevent* OR control OR management OR treat*
4	Barriers	barrier* OR challenge* OR lack* OR "pharm* barrier*" OR "patient* barrier*" OR "policy* barrier*" OR "health?care* barrier*"
5	Ethiopia	"*Ethiopia*"
Medline (Ovid)<br>1 and 2 and 3 and 4 and 5 and limit to English language		(Pharm* OR "Communit* pharm*" OR "communit* pharm* profession*" OR "communit* drug retail*" OR "drug retail*" OR "communit* medic* retail*" OR CPPs) AND ("non?commun* diseases" OR "chronic* disease*" OR "non?communicable*ADJ3 disease*" OR "cardiovascular disease*" OR hypertension OR "heart failure" OR "metabolic syndrome" OR diabetes OR "chronic* respiratory disease*" OR asthma OR "chronic* obstructive pulmonary disease*" OR cancer OR NCDs) AND (role* OR practices OR involvements OR counseling OR dispensing OR "health promotion*" OR "health education" OR "lifestyle modification" OR "medic* therapy management" OR "medic* adherence" OR "chronic* patient* care*" OR screen* OR monitor* OR prevent* OR control OR management OR treat*) AND (barrier* OR challenge* OR lack* OR "pharm* barrier*" OR "patient* barrier*" OR "policy* barrier*" OR "health?care* barrier*") AND ("Ethiopia") {Including Related Terms} AND<br>limit 2 to English language

**Scopus**<br>1 and 2 and 3 and 4 and 5 and limit to English language	TITLE-ABS-KEY ((pharm* OR "Communit* pharm*" OR "communit* pharm* profession*" OR "communit* drug retail*" OR "drug retail*" OR "communit* medic* retail*" OR cpps) AND ("non?commun* diseases" OR "chronic* disease*" OR "non?communicable*ADJ3 disease*" OR "cardiovascular disease*" OR hypertension OR "heart failure" OR "metabolic syndrome" OR diabetes OR "chronic* respiratory disease*" OR asthma OR "chronic* obstructive pulmonary disease*" OR cancer OR ncds) AND (role* OR practices OR involvements OR counseling OR dispensing OR "health promotion*" OR "health education" OR "lifestyle modification" OR "medic* therapy management" OR "medic* adherence" OR "chronic* patient* care*" OR screen* OR monitor* OR prevent* OR control OR management OR treat*) AND (barrier* OR challenge* OR lack* OR "pharm* barrier*" OR "patient* barrier*" OR "policy* barrier*" OR "health?care* barrier*") AND ("Ethiopia")) AND (LIMIT-TO (LANGUAGE , "English"))
