# Supplementary material for: Roles and barriers of community pharmacy professionals in the prevention and management of noncommunicable diseases in Ethiopia: a systematic review
Source: Front Public Health. 2025 Aug 28;13:1485327. doi: 10.3389/fpubh.2025.1485327 (PMC12422921; doi:10.3389/fpubh.2025.1485327)
Supplement: Supplementary file 3 [file Data_Sheet_3.pdf]

Quality Assessment of the studies

Studies	Clear inclusion criteria	Description of study subject and study setting	A valid and reliable method to measure the exposure	Standard criteria used for measurement of the condition	Identification of confounding factors	Develop strategies to deal with confounding factors	A valid and reliable method to measure outcomes	Appropriate statistical analysis	Total score out of 8	Level of bias
Belachew et al., 2024	Yes	Yes	Yes	Yes	Yes	N/A	Yes	Yes	7	Low
Sendekie et al., 2024	Yes	Yes	Yes	Yes	N/A	N/A	Yes	Yes	6	Low
Sendekie et al., 2023	Yes	Yes	Yes	Yes	Yes	N/A	Yes	Yes	7	Low
Sendekie et al., 2023	Yes	Yes	Yes	Yes	Yes	N/A	Yes	Yes	7	Low
Sendekie et al., 2023	Yes	Yes	Yes	Yes	N/A	N/A	Yes	Yes	6	Low
Ayenew et al., 2022	Yes	Yes	Yes	Yes	N/A	N/A	Yes	Yes	6	Low
Birarra et al., 2022	Yes	Yes	Yes	Yes	Yes	N/A	Yes	Yes	7	Low
Sendekie et al., 2022	Yes	Yes	Yes	Yes	N/A	N/A	Yes	Yes	6	Low
Emiru et al., 2020	Yes	Yes	Yes	Yes	N/A	N/A	Yes	Yes	6	Low
Belachew et al., 2020	Yes	Yes	Yes	Yes	Yes	N/A	Yes	Yes	7	Low
Moges et al., 2019	Yes	Yes	Yes	Yes	N/A	N/A	Yes	Yes	6	Low
Teka et al., 2018	Yes	Yes	Yes	Yes	N/A	N/A	Yes	Yes	6	Low
Eriku et al., 2017	Yes	Yes	Yes	Yes	Yes	N/A	Yes	Yes	7	Low
Asmelashe et al., 2017	Unclear	Yes	Yes	Yes	Yes	N/A	Yes	Yes	6	Low
Eriku et al., 2017	Yes	Yes	Yes	Yes	Yes	N/A	Yes	Yes	7	Low

Moola S, Munn Z, Tufanaru C, Aromataris E, Sears K, Sfetcu R, Currie M, Qureshi R, Mattis P, Lisy K, Mu P-F. Chapter 7: Systematic reviews of etiology and risk. In: Aromataris E, Munn Z (Editors). *JBI Manual for Evidence Synthesis*. JBI, 2020. Available from https://synthesismanual.jbi.global
